# Role of *Leishmania (Leishmania) chagasi *amastigote cysteine protease in intracellular parasite survival: studies by gene disruption and antisense mRNA inhibition

**DOI:** 10.1186/1471-2199-6-3

**Published:** 2005-02-03

**Authors:** Vasanthakrishna Mundodi, Ashwini S Kucknoor, Lashitew Gedamu

**Affiliations:** 1 Department of Biological Sciences, University of Calgary, Calgary AB T2N1N4, Canada

## Abstract

**Background:**

The parasitic protozoa belonging to *Leishmania (L.) donovani *complex possess abundant, developmentally regulated cathepsin L-like cysteine proteases. Previously, we have reported the isolation of cysteine protease gene, *Ldccys2 *from *Leishmania (L.) chagasi*. Here, we have further characterized this cysteine protease gene and demonstrated its role during infection and survival of *Leishmania (L.) chagasi *within the U937 macrophage cells.

**Results:**

The amastigote specific Ldccys2 genes of *L. (L.) chagasi *and *L. (L.) donovani *have identical gene organization, as determined by southern blots. In *vivo *expression analyses by Northern blots showed that *Ldccys2 *is amastigote specific. Western blot using anti-Ldccys2 antibody confirmed the amastigote specific protein expression. Recombinant expression of Ldccys2, a 30 kDA protein, was functionally active in a gelatin assay. Results from *Ldccys2 *heterozygous knockout mutants showed its role during macrophage infection and in intra-macrophage survival of the parasites. Since attempts to generate null mutants failed, we used antisense RNA inhibition to regulate *Ldcccys2 *gene expression. Not surprisingly, the results from antisense studies further confirmed the results from heterozygous knockout mutants, reiterating the importance of amastigote specific cysteine proteases in *Leishmania *infection and pathogenesis.

**Conclusions:**

The study shows that *Ldccys2 *is a developmentally regulated gene and that Ldccys2 is expressed only in infectious amastigote stages of the parasite. The collective results from both the heterozygous knockout mutants and antisense mRNA inhibition studies shows that Ldccys2 helps in infection and survival of *L. (L.) chagasi *amastigotes within the macrophage cells. Finally, antisense RNA technique can be used as an alternate approach to gene knockout, for silencing gene expression in *L. (L.) chagasi*, especially in cases such as this, where a null mutant cannot be achieved by homologous recombination.

## Background

*Leishmania *are the etiological agents of a variety of disease manifestations, collectively termed as leishmaniasis. Visceral leishmaniasis caused by *Leishmania (L.) donovani *and *Leishmania (L.) chagasi *is a serious health problem in many tropical and subtropical countries [1-3]. During the digenetic life cycles of *Leishmania*, it alternates between gut of sand fly vector as an extra cellular promastigote and in the acidic phagolysosome of macrophage as an intracellular amastigote. However, the most intriguing question is the capacity of *Leishmania *to withstand the hydrolytic conditions of the macrophages, the mechanism of which is still unclear. Thus, identifying the genes expressed specifically in the amastigote stage of the parasites and elucidating their biological function is very important as it would provide new insights into the role of these gene products in the intracellular life cycle of the parasites. Further, this will also help in designing specific drugs and identifying vaccine candidates.

Cysteine proteases play an important role in the infection, replication, development and metabolism of protozoan parasites [4,5]. They have been implicated in the invasion of human erythrocyte by *Plasmodium falciparum *[6] and considered as virulence factors in the pathogenesis of *Entamoeba histolytica *[7]. Cysteine protease activity is necessary for the survival of *Leishmania (L.) mexicana *[8,9] and related protozoan, *Trypanosoma cruzi*, within the macrophages, *in vitro *[10]. Knockout studies in *L. (L.) mexicana *have shown that cysteine proteases not only are virulence factors but also act as modulators of host immune responses [11,12]. Thus, cysteine proteases have become a potential target for chemotherapy and a candidate for vaccine development. Initial studies have confirmed the efficacy of cysteine protease inhibitors in treatment of *T. cruzi*, *P. falciparum *and *L. (L.) major *[13-15]. Immunization with the hybrid protein vaccine, consisting of *L. (L.) major *cysteine proteases CPB and CPA, partially protected against leishmaniasis [16]. So far, functionally well characterized cysteine proteases are from the New World species of *Lesihamania *causing the cutaneous forms of leishmaniasis. The members of *L .(L.) donovani *complex also possess multiple classes of cysteine proteases, which are developmentally regulated [17,18] and are not functionally well characterized. Therefore, there is a need to study the function of these proteases and their role in visceral leishmaniasis.

Studies aimed at defining the function of protozoan parasite components have often used gene disruption approach by homologous recombination. Recently an alternative antisense RNA method was followed that may easily and rapidly answer the complex biological questions. Anti sense RNA approach has been used to study the functions of certain gene products in *Entamoeba *and *Leishmania*, to elucidate the functions of cysteine protease [19,20], A2 protein [21] and gp63 [22]. Previously, we have isolated and characterized two distinct cysteine protease cDNA clones *Ldccys1 *and *Ldccys2 *from promastigote and amastigote specific cDNA libararies of *L. (L.) chagasi *[17]. *Ldccys1*, a member of multi gene family was characterized both in *L. (L.) chagasi *(*Ldccys1*) and *L. (L.) donovani *(*Lddcys1*) parasites [18]. In the present study, we have characterized the functional role of amastigote specific cysteine protease gene (*Ldccys2*) of *L. (L.) chagasi*. We have generated *Ldccys2 *heterozygous knockout mutants of *L. (L.) chagasi *by homologous recombination. As an alternative approach, antisense mRNA expression was also employed. Results obtained from both, gene disruption and antisense mRNA expression shows that Ldccys2 plays an important role in the survival of amastigotes within the U937 macrophages.

## Results

### Ldccys2 is a single copy gene

**Figure 1:**
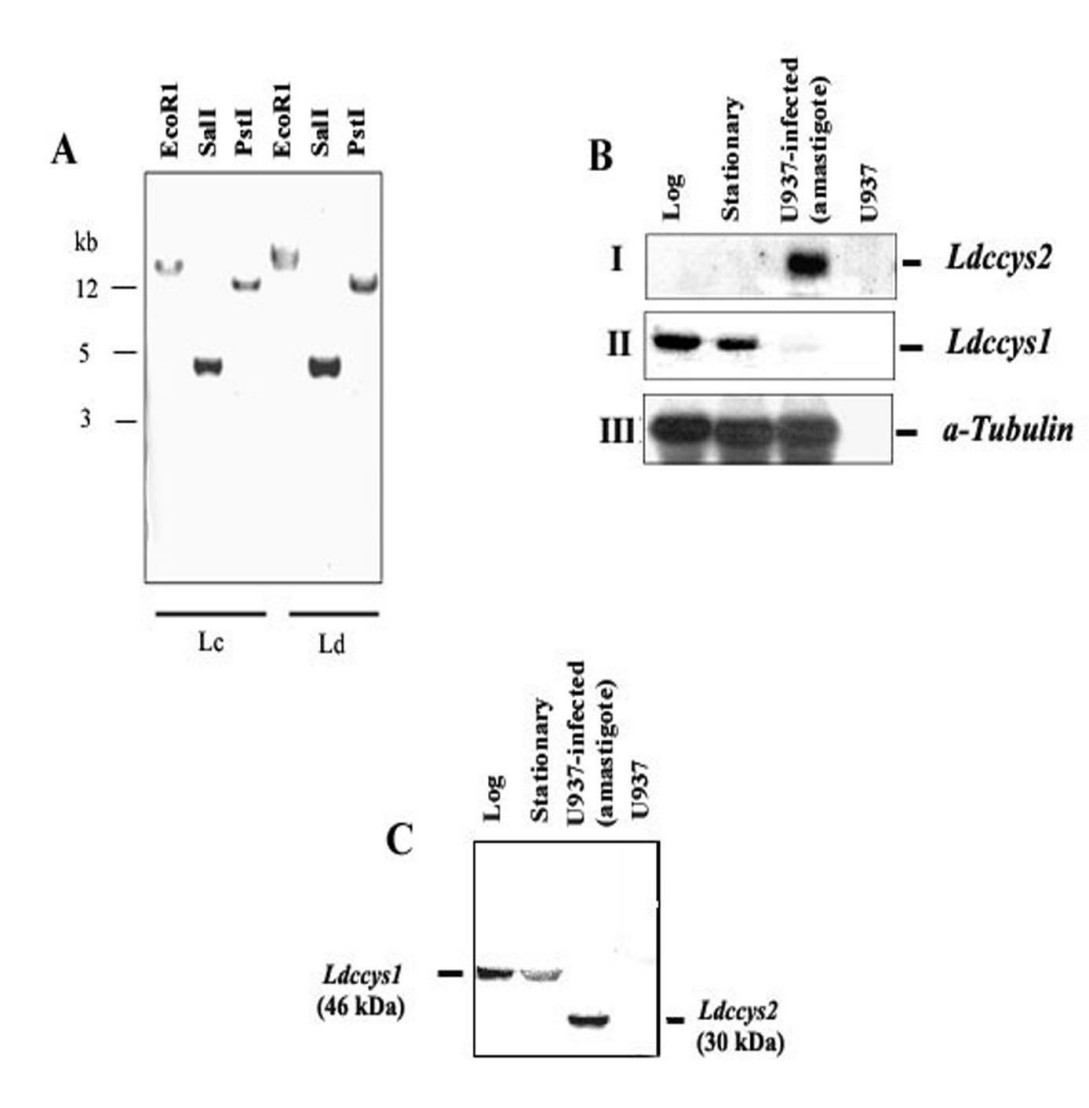
***Ldccys2 *is a single copy gene and is expressed only in amastigotes. **(A) Southern blot hybridization of digested genomic DNA from *L. (L.) chagasi *(Lc) and *L. (L.) donovani *(Ld). 5 μg of genomic DNA was digested with restriction enzymes as mentioned in the figure and blotted onto Hybond-N membranes. The blot was probed with coding region pf *Ldccys2 *cDNA clone. (B) Northern blot analyses of *L. (L.) chagasi *total RNA. Total RNA (10 μg/lane) from promastigotes of logarithmic (lane 1), stationary (lane 2) growth phase, U937 cells (human macrophage cell line) infected with promastigotes for 96 h (lane 3) and uninfected U937 cells (lane 4) were separated on 1.2% (w/v) formaldehyde agarose gel and transferred on to Hybond N+ membrane. Blot was hybridized with PCR amplified DNA fragment containing 3'UTR from *Ldccys2 *(panel I), *Ldccys1 *(154 bp, near the polyA region, panel II), and coding region of α-*tubulin *from *L. (L.) chagasi*, a kind gift from Dr. M.E. Wilson (panel IV). (C) Western blot analysis of *L. (L.) chagasi *promastigotes and amastigotes. Equal amounts of proteins from promastigotes of logarithmic (lane 1), stationary (lane 2) phase, U937 cells infected with promastigotes (lane 3) and uninfected U937 cells (lane 4) were separated on 10% (w/v) SDS-PAGE and blotted on to Hybond – P membrane. Western blot analysis was carried out using α-Ldccys2 antibody.

From our earlier studies it is known that *Ldccys2 *is a single copy gene in *L. (L.) chagasi*. In order to compare the genomic organization of *Ldccys2 *gene in *L. (L.) donovani and L. (L.) chagasi*, the members of *L. (L.) donovani *complex, Southern analysis of genomic DNA was performed. Genomic DNA was digested with different restriction enzymes as shown in Figure 1A and probed with *Ldccys2 *coding region as a probe. Identical hybridizing bands were present in both *L. (L.) chagasi *and *L. (L.) donovani *(Figure 1A). PCR amplification and sequencing of *Ldccys2 *coding region from *L. (L.) donovani *genomic DNA showed identical nucleotide sequences, indicating that *L. (L.) chagasi *and *L. (L.) donovani *had identical *Ldccys2 *cysteine protease genes.

### Ldccys2 gene is differentially expressed

To study the expression of *Ldccys2 *cysteine protease genes, Northern blot analysis was performed using *L. (L.) chagasi *total RNA from log and stationary phase promastigotes and amastigotes. The 3'UTR region of *Ldccys2 *used as a probe, hybridized to a 2.5 kb transcript in amastigote (Figure 1BI) unlike the cysteine protease, *Ldccys1*, which is predominantly expressed in promastigote stages of the parasite (Figure 1 BII). The α-*tubulin *gene expression was used as an internal control (Figure 1 BIII). *Ldccys2 *showed similar pattern of expression in *L. (L.) donovani *(data not shown). It is important to note that this result is in contrast to the earlier report (17), wherein *Ldccys2 *was identified as promastigote specific cysteine protease gene and *Ldccys1 *was thought to be amastigote specific. We have confirmed the expression pattern and rectified this error in the following publication (18).

To further confirm the amastigote expression of Ldccys2, Western blot analysis was carried out using extracts from both promastigotes and U937 infected amastigotes of *L. (L.) chagasi*. The anti-Ldccys2 antibody detected a 30 kDa band in amastigotes alone (Figure 1C, lane 3).

However, a single 46 kDa band seen in log and stationary phase promastigotes (Figure 1C, lanes 1 and 2) represents promastigote specific cysteine protease Ldccys1, due to the cross-reactivity of the antibody. Taken together, the above results show that Ldccys2 is expressed only in the amastigote stage of *L. (L.) chagasi*.

### Recombinant Ldccys2 expressed in insect cells is functionally active

**Figure 2:**
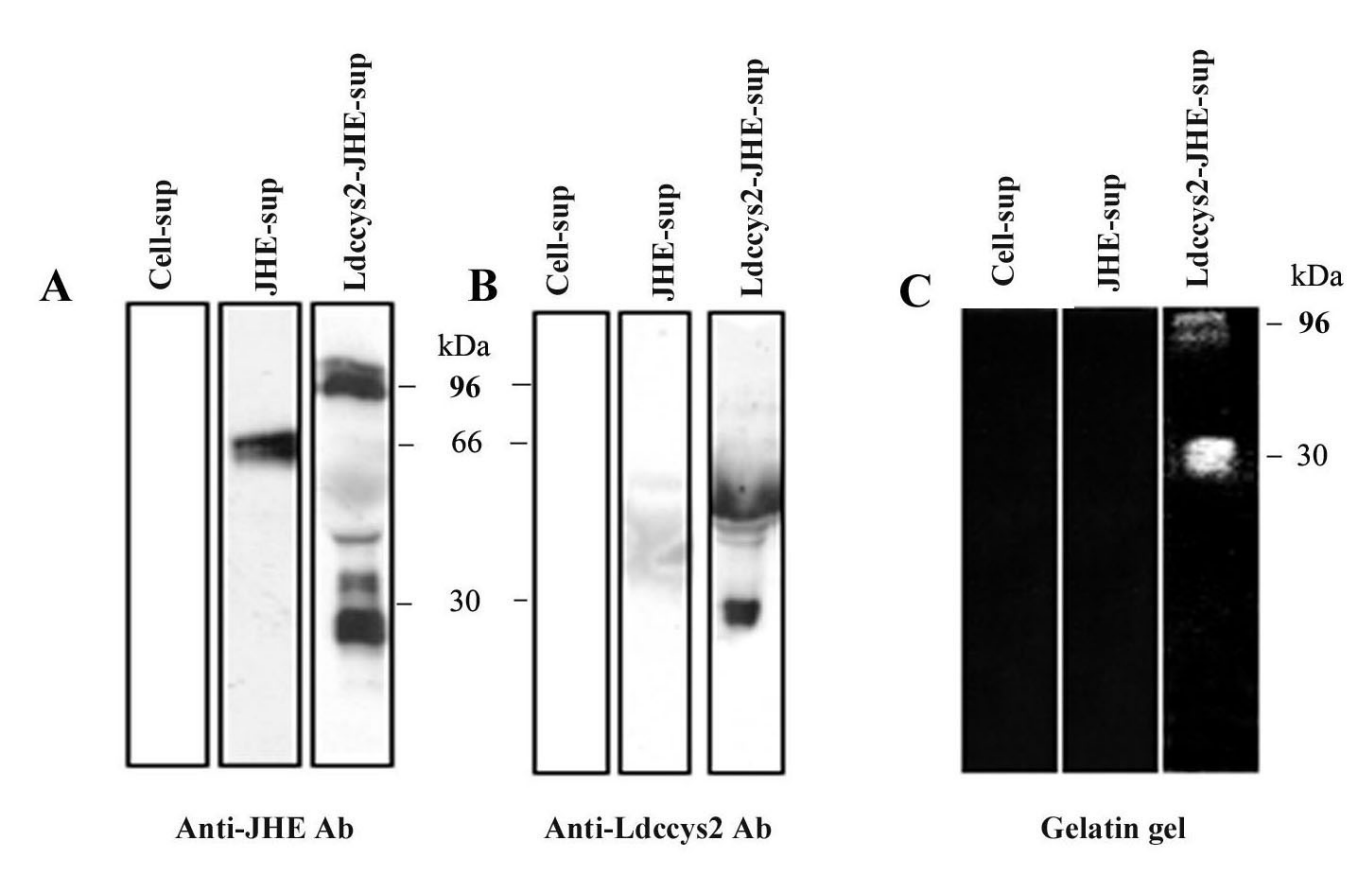
**Expression of recombinant Ldccys2 using insect cell expression system. **Western blots showing recombinant expression of Ldccys2 using anti-JHE antibody (A) and duplicate blot probed with anti-Ldccys2 antibody (B). The lanes are labeled appropriately. 5 μg of supernatant from the insect cells expressing recombinant plasmids or the control were separated on 8% SDS-PAGE, blotted onto to PVDF membranes and probed with 1:500 dilution of anti-JHE or 1:100 dilution of anti-Ldccys2 antibody. A horseradish peroxidase conjugated anti-mouse secondary antibody was used and the blot was developed by using ECL chemiluminescent kit. (C) Gelatin assay showing cysteine protease activity of the recombinant protein. The supernatant was separated on a gelatin gel and incubated in a reducing buffer. The gel was then stained with Coomassie Blue to visualize the clearing of gelatin on the gel indicating cysteine protease activity.

In order to determine the cysteine protease activity encoded by *Ldccys2*, the predicted coding region was cloned into pIE1/153A.jhe (6hep) vector and transfected into High Five insect cells (invitrogen). The supernatant was harvested and analyzed for the presence of recombinant protein by Western blot analysis using JHE and Ldccys2 antibodies. Western blot analyses with α-JHE antibody (Figure 2A, lane3) detected a band of 96 kDa, corresponding to the fusion protein. The lower molecular mass bands are due to the processed products as a result of autocatalytic activity. Anti- Ldccys2 antibody however, could not detect the recombinant fusion protein (Figure 2B lane 3). The processed products were detected instead, which may be because of inaccessibility of the epitope in the fusion protein. Both the antibodies did not give any background with the negative control (cell extract, Figure 2B and 2C, lane1). Protease activity determined by gelatin SDS-PAGE, showed a band of approximately 30 kDa (Figure 2C, lane 3), confirming the protease activity. Cysteine protease activity was completely inhibited by a specific inhibitor, E-64 at 20 μM concentration (data not shown).

### Ldccys2 single allele gene replacement by homologous recombination

**Figure 3:**
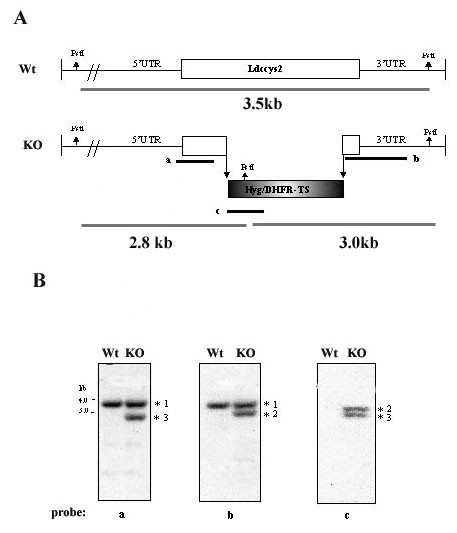
***Ldccys2 *single allele gene replacement. **(A) Schematic representation of the wild type and *hyg/dhfr-ts *targeted alleles of *Ldccys2*. The location of the *Pst*I sites used to characterize the hygromycin B resistant recombinants is shown by arrows. The bold lines labelled a, b and c represents the location of different probes used in Southern analyses. (B) Southern analyses of *Ldccys2 *heterozygous knockout mutants. Genomic DNA (2 μg/lane) from wild type *L. (L.) chagasi *(WT) and *Ldccys2 *heterozygous knockout mutants (KO) were digested with *Pst*I and separated on a 0.9% (w/v) agarose gel. The DNA was blotted on to Hybond N+ membrane and hybridized with probes a (i), b (ii) and c (iii). Asterisks: 1- represents wild type allele of *Ldccys2 *(3.5 kb), 2 and 3- represent disrupted allele (3.0 kb and 2.8 kb). The grey lines indicate the size of expected bands upon PstI digestion.

In order to investigate the function *Ldccys2 *gene, we have performed gene replacement by disrupting a 500 bp coding region of the gene with *hyg *and *dhfr-ts *cassette as shown in figure 3A. Following transfection, hygromycin B resistant parasites (*Ldccys2KO*, heterozygous knockout mutants) were selected for further analysis. The *Pst*I restriction enzyme digested genomic DNA from wild type *L. (L.) chagasi *and *Ldccys2 KO *were subjected to Southern analysis and results are presented in figure 3B. The disrupted *Ldccys2 *allele had an additional *Pst*I site that is present within the *hyg *gene. As shown in the figure 3B, 5' and 3'probes (probes a and b) hybridized to a single 3.5 kb band with wild type genomic DNA. In *Ldccys2KO*, probe-a hybridized to two bands (3.5 kb and 2.8 kb) and probe-b hybridized to 3.5 kb and 3.0 kb bands. As expected, one of the bands corresponds to the wild type allele whereas the other band corresponded to the disrupted allele. The *hyg *specific probe-c resulted in no hybridizing band with *Pst*I digested wild type genomic DNA, while in *Ldccys2KO*, two bands (2.8 kb and 3.0 kb) corresponding to the disrupted allele were detected (Figure 3B, probe c). These results clearly indicate that one of the alleles of the *Ldccys2 *gene has been replaced by hygromycin gene. Similarly, a second cassette using neomycin gene was used in order to replace the second allele of *Ldccys2 *gene. Despite numerous attempts, we were unable to obtain a null mutant of *Ldccys2*. We then checked for any events of rearrangements or change in ploidy levels in the heterozygous mutants. No such detectable events were detected (data not shown).

### Ldccys2 single allele knock-out mutants had reduced mRNA and protein levels

**Figure 4:**
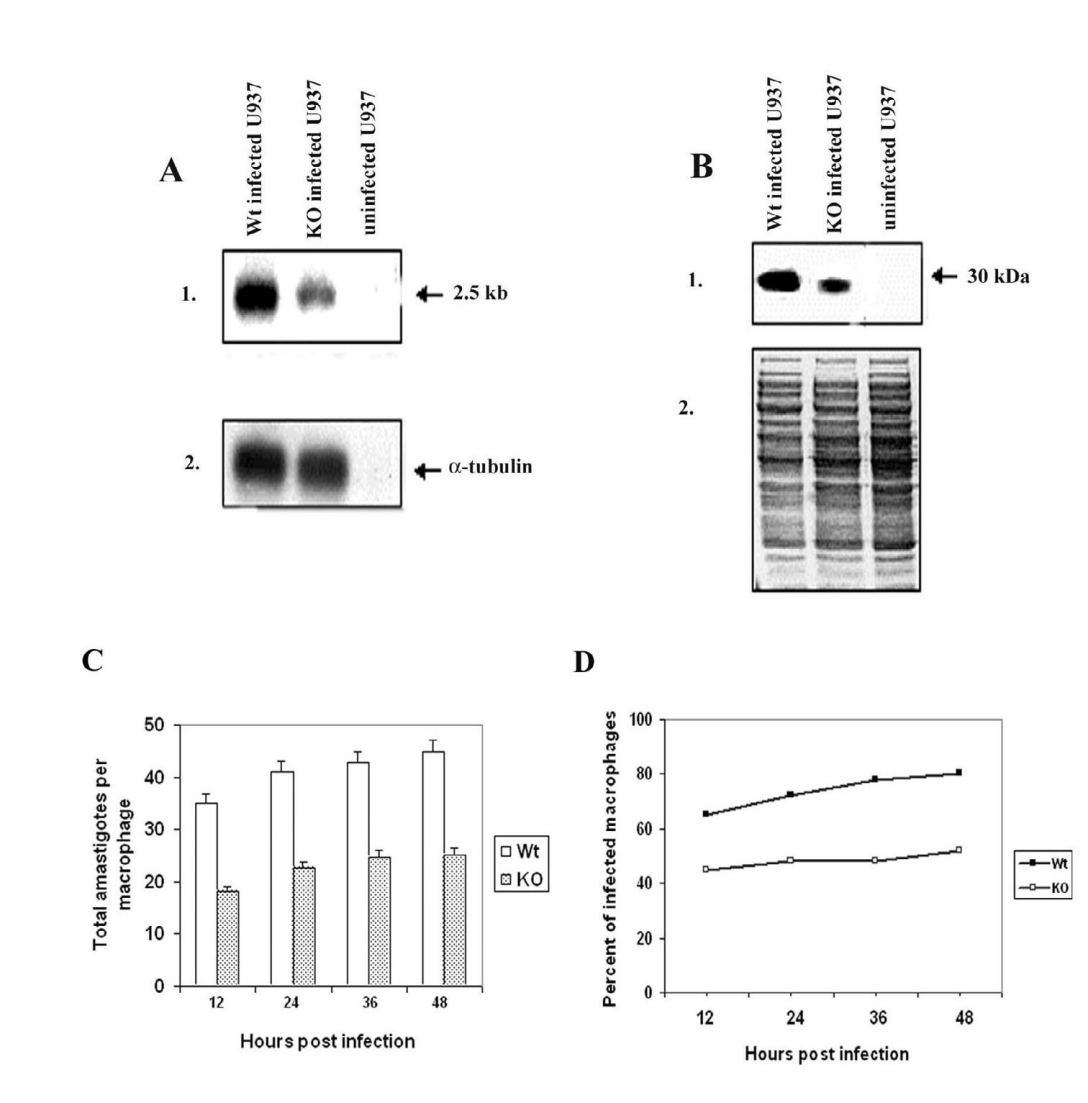
**Characterization of *L. (L.) chagasi *heterozygous knockout mutant amastigotes and intra macrophage survival of *Ldccys2KO *amastigotes *in vitro*. **(A) Northern blot analysis. Total RNA (10 μg/lane) from U937 cells infected with wild type promastigotes, *Ldccys2KO *and uninfected U937 cells were separated on 1.2% (w/v) formaldehyde agarose gel and transferred on to Hybond N+ membrane. Blot was hybridized with *Ldccys2 *coding region DNA probe (1) and α-tubulin gene from *L. (L.) chagasi *(2). (B) Western blot analysis. Equal amount of lysates from U937 cells infected with wild type promastigotes, *Ldccys2KO *promastigotes and uninfected U937 cells were separated on 10% (w/v) SDS-PAGE, blotted on to Hybond P membrane. Panel 1 represents the membrane that was probed with α-Ldccys2 antibody and panel 2 is the duplicate gel stained with Coomassie blue. (C) Bar graph showing the number of intracellular amastigotes. U937 macrophage cells were infected at a macrophage to parasite ratio of 1:10. The survival of the amastigotes within the macrophages was evaluated every 12 hours by cytospin and Diff-Quick staining. (D) Graph showing the percent of total macrophages infected at the given time points. For each treatment, 100 infected macrophages were counted. Values represent means ± SEM from three independent experiments.

In order to compare the expression of *Ldccys2 *gene in wild type and heterozygous knockout mutants (*Ldccys2 KO*), amastigote RNA was isolated from U937 cells infected with wild type as well as *Ldccys2KO *mutant *L. (L.) chagasi *promastigotes. Northern blot analysis with Ldccys2 coding region probe detected a single transcript of 2.5 kb from wild type as well as *Ldccys2 KO *amastigotes (Figure 4A, panel 1). However, the transcript level of *Ldccys2 *in the *Ldccys2KO *amastigotes was reduced by 64% compared to that in the wild type *L. (L.) chagasi*. There was no change in the level of α-tubulin expression either in the *Ldccys2KO *or in the wild type amastigotes (Figure 4A, panel 2).

The effect of *Ldccys2 *single allele disruption at protein level was studied using western blot analysis. Equal amounts of lysates from wild type and *Ldccys2KO L. (L.) chagasi *amastigotes were analyzed using antiserum raised against Ldccys2 protein. Wild type *L. (L.) chagasi *amastigotes showed a major band of 30 kDa (Figure 4B, panel 1), while *Ldccys2KO *amastigotes showed an identical sized band, but there was a reduction in amounts by 45%, based on the quantitation of bands. The antibody did not pick up any signal from the negative control (U937 cells). Figure 4B, panel 2 indicates the equal loading of proteins. These results once again, confirm the deletion of one of the alleles of *Ldccys2*.

### Ldccys2 heterozygous knockout mutants exhibit reduced infectivity and intra-macrophage survival of amastigotes

In order to study the effect of Ldccys2 heterozygous knockout mutants on parasite growth, a growth curve analysis was performed. No significant differences were observed between the wildtype parasites and the *Ldccys2KO *(data not shown). Next, we performed an infection assay with differentiated U937 macrophage cells using with wild type and *Ldccys2KO L. (L.) chagasi *promastigotes. Interestingly, twelve hours after infection, the number of amastigotes in *Ldccys2KO *infected macrophages were reduced by half as compared to that of wild type infected macrophage cells. This trend remained same at all the time intervals tested (24, 36 and 48 hours post infection), indicating that the intracellular amastigotes were not able to multiply efficiently (Figure 4C). The percentage of infected macrophages in the wildtype and *Ldccys2KO *were similar at 12, 24, 36 and 48 hours post infection (Figure 4D), however, there was a clear decrease in the initial infection in case of *Ldccys2KO*, as compared to the wildtype parasites. The above data suggests that Ldccys2 heterozygous knockout has resulted in reduced levels of Ldccys2 expression and furthermore, has decreased the ability of the amastigotes to infect the macrophages and survive inside the macrophage cells. Taken together, these results suggest that Ldccys2 gene has a significant role in the infection and intramacrophage survival of amastigotes.

### Antisense mRNA inhibition of Ldccys2 resulted in reduced macrophage infection and poor amastigote survival within the macrophages

**Figure 5:**
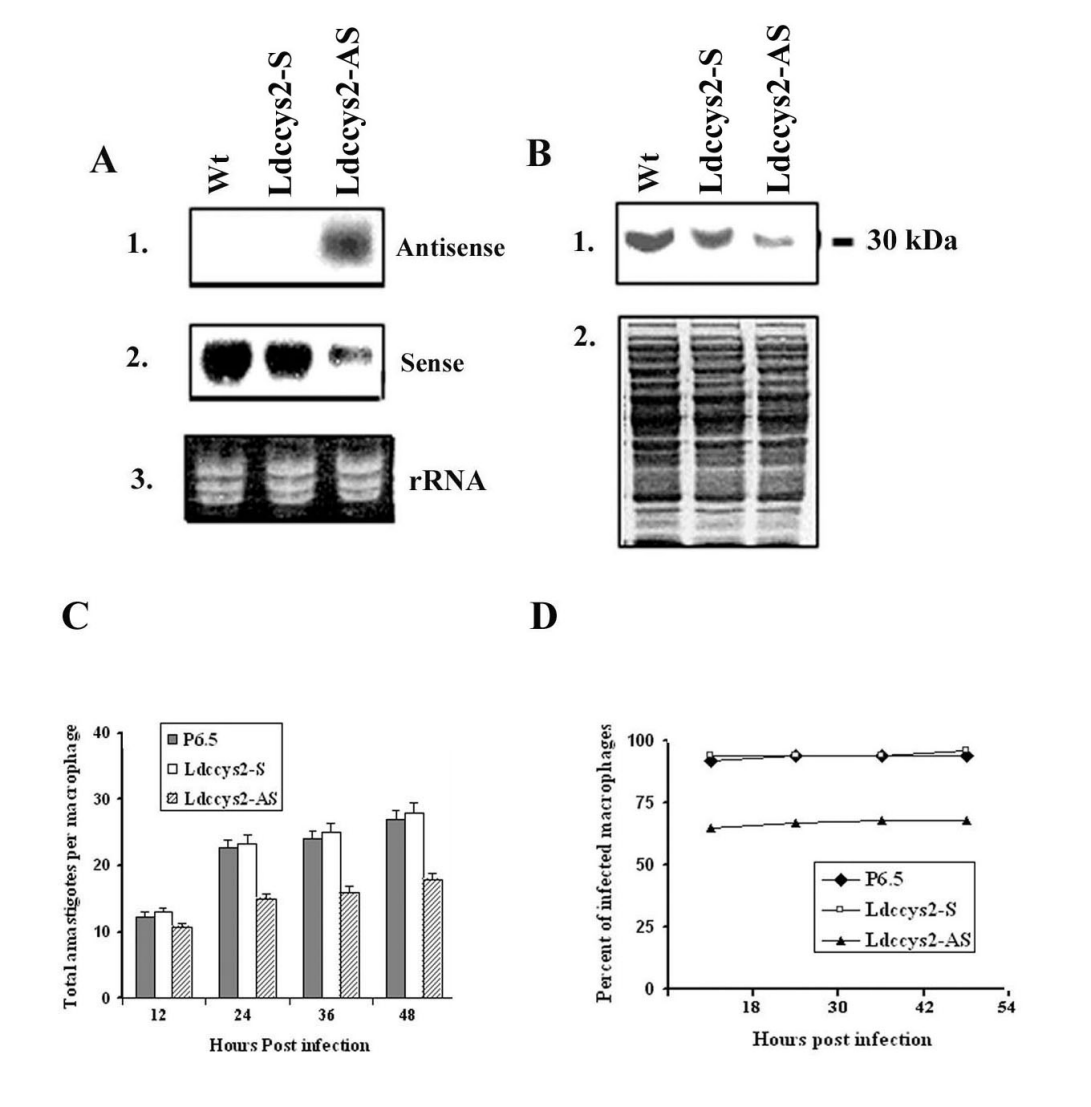
**Expression of sense and antisense *Ldccys2 *transcripts in *L. (L.) chagasi *and survival of wild type, sense and antisense *Ldccys2 *expressing amastigotes in macrophages. **(A) Northern blot analyses of amastigotes expressing sense and antisense transcripts. Total RNA (10 μg/lane) isolated from axenic amastigotes of wildtype parasites, transfectants with P6.5/Ldccys2 sense plasmid and P6.5/Ldccys2 antisense plasmid were separated on 1.2% (w/v) formaldehyde agarose gel and blotted on to Hybond N+ membrane. Blots were probed with probes specific for antisense (panel 1) and sense (panel 2) transcripts. Panel 3 represents ethidium bromide stained gel. (B) Western blot analysis of sense and antisense Ldccys2 expressing amastigotes. Equal amounts of total protein extracted from axenically transformed amastigotes with P6.5/Ldccys2 sense plasmid and P6.5/Ldccys2 antisense plasmid and wildtype parasites were separated on 10% (w/v) SDS-PAGE and blotted on to Hybond P membrane. The blot was probed with α-Ldccys2 antibody (panel 1) and a duplicate gel was stained with Coomassie blue (panel2). (C) Bar graph showing total number of amastigotes within the macrophage cells. U937 macrophage cells were infected at a macrophage to parasite ratio of 1:10. The survival of the amstigotes within the macrophages was evaluated every 12 hours by cytospin and Diff-Quick staining.(D) Graph showing the percent of total macrophages infected at the given time points. For each treatment, 100 infected macrophages were counted. Values represent means ± SEM from three independent experiments.

We were unable to generate a complete knock out mutant of *Ldccys2 *and the heterozygous knockout mutants only partially inhibited the gene expression. Therefore, we used an alternative approach of antisense mRNA inhibition to silence the *Ldccys2 *gene and to confirm the conclusions derived from *Ldccys2 *heterozygous knockout mutants studies. Plasmid constructs containing the *Ldccys2 *antisense and sense sequences were transfected into *L. (L.) chagasi *promastigotes. The presence of plasmids in these transfectants was confirmed by Southern analysis with *Ldccys2 *probes (data not shown). Northern blot analyses of amastigotes expressing different plasmid constructs were carried out using specific sense and antisense probes. As expected, *Ldccys2 *antisense mRNA was expressed only in amastigotes with antisense constructs (Figure 5A, panel 1), and these amastigotes showed a 74% decrease in the *Ldccys2 *mRNA levels (Figure 5A, panel 2). The amastigotes containing either the sense plasmid or the control plasmid did not show any change in the levels of *Ldccys2 *mRNA (Figure 5A, panel 2). The stained agarose gel indicates the equal amounts of RNA used in the Northern blots (Figure 5A, panel 3). These results clearly demonstrate that the episomal expression of *Ldccys2 *antisense mRNA reduces the levels of endogenous *Ldccys2 *mRNA.

We next performed Western blot analysis of transfectants expressing sense and antisense *Ldccys2 *mRNA using α-Ldccys2 antibody. Amastigotes expressing antisense mRNA showed a significant reduction (by 76%) in the levels of Ldccys2, however, the expression of sense mRNA did not increase the levels of Ldccys2 compared to amastigotes containing the control plasmid (Figure 5B, panel1). Equal loading of the protein is depicted by the Coomassie stained gel (Figure 5B, panel 2). The results further confirm that *Ldccys2 *antisense mRNA expression does inhibit the endogenous levels of Ldccys2.

Survival of *L. (L.) chagasi *amastigotes within the macrophages was assessed *in vitro*, using U937 cells. Results from three individual experiments are pooled and the collective data is presented in figure 5C. Results showed that the amastigotes carrying either the control plasmid (P6.5) or expressing *Ldccys2 *sense mRNA did not have any effect on their capacity to infect and survive inside the macrophages. The number of amastigotes per macrophage cell remained the same at initial stages of infection (data not shown). However, amastigotes expressing *Ldccys2 *antisense mRNA showed significant reduction (by almost 50%) in terms of the initial infection and intra macrophage survival starting from 12 hours post infection and remained consistent up to 48 hours after infection (Figure 5D). The percentage of total infected macrophages is shown in figure 5D. Consistent with the results from the heterozygous knockout mutants, the *Ldccys2 *antisense transfectants had reduced levels of infection when compared to that of the wildtype and control plasmids. These results further underline the importance of Ldccys2 in infection of macrophages and subsequent survival of amastigotes within the macrophage cells. Taken together, these results indicate that the amastigote specific cysteine protease, Ldccys2 plays an important role in intramacrophage survival of *L. (L.) chagasi *amastigotes.

## Discussion

In this study, we have shown that amastigote cysteine protease, Ldccys2 necessary for macrophage infection and for survival of *L. (L.) chagasi *amastigotes inside the macrophage cells using two different approaches, gene disruption and antisense mRNA expression. *Leishmania (L.) chagasi *cysteine protease, *Ldccys2 *is a single copy gene [18] and is expressed only in amastigote stage of the parasite. *L. (L.) donovani *also possesses an identical gene. Expression studies performed demonstrates that *Ldccys2 *is expressed only in amastigotes of *L. (L.) chagasi *and *L. (L.) donovani*. A similar single copy gene in *L. (L.) mexicana *(*Lmcpa) *has a significant sequence identity (80%) to *Ldccys2*. *Lmcpa *gene of *L. (L.) mexicana *which is the closest homolog of *Ldccys2 *is differentially expressed in amastigotes [23], whereas the *Ldccys2 *gene of *L. (L.) chagasi *is expressed specifically in the amastigote stage. In addition, the major amastigote specific cysteine proteinase gene of *L. (L.) mexicana*, *cpb2.8 *is a multicopy gene, located within the *lmcpb *cluster [24] and there is no significant sequence identity (34%) between *Ldccys2 *and *lmcpb2.8*. Therefore, gene organization and expression of amastigote specific cysteine proteases in *L. (L.) mexicana *and *L. (L.) donovani *complex are not identical. Interestingly, *Llacys1*, an amastigote specific cysteine protease from *L. (L.) amazonensis *was identified recently, which has high homology (88%) with *Ldccys2 *and like *Ldccys2*, is only expressed in amastigote stages [25]. In order to confirm the protease activity of amastigote cysteine protease, full-length *Ldccys2 *was expressed as JHE-fusion protein in an insect cell expression system. The recombinant protein hydrolysed gelatin in activity gels.

Recently, functions of many genes have been established in *Leishmania *by following gene disruption approach [26,27]. To study the biological role of Ldccys2 cysteine protease, we have followed targeted gene disruption using *hyg *gene as the selectable marker. Genomic Southern analyses of the hygromycin B resistant clones using specific probes confirmed the replacement of 500 bp region within the ORF of the *Ldccys2 *by *hyg/dhfr-ts*. Northern and Western blot analyses *Ldccys2KO *amastigotes showed a clear decrease in the endogenous levels of Ldccys2 compared to that of wild type *L. (L.) chagasi *amastigotes (Figure 4), demonstrating the disruption of one of the *Ldccys2 *alleles. Despite our repeated effort using different selectable marker genes, we failed to create a null mutant of *Ldccys2 *in *L. (L.) chagasi*. Since gene amplification and increase in ploidy level has been reported for *Leishmania *species [28], we analyzed for changes in ploidy level and or gene amplification and found no such events occurring in these heterozygous knockout mutants. This suggests that *Ldccys2 *is an important gene necessary for the initiation of macrophage infection and survival of amastigotes within the macrophages. Therefore we used the *Ldccys2 *heterozygous knockout parasites for further functional studies. The U937 macrophage cells infected with *Ldccys2 *heterozygous knockout mutants showed a significant decrease in the initial infection and survival within the macrophage cells, compared to wild type (Figure 4). This trend remained consistent from 12 hours up to 48 hours after infection. The decrease in initiation of infection and subsequent survival of amastigotes may explain the role of Ldccys2 in amastigote metabolism and its survival inside the macrophages [4]. Consistent with our results, null mutants of the *lmcpb *cluster in *L. (L.) mexicana *exhibited reduction in macrophage infectivity and survival [23]. However, lack of *Ldccys2 *null mutant prevented us from a detailed study on the role of Ldccys2 in survival and pathogenesis, using animal models.

The lack of *Ldccys2 *null mutants prompted us to use an alternative approach of antisense mRNA inhibtion in order to confirm the results obtained from *Ldccys2 *heterozygous knockout studies. Using antisense RNA technique it has become possible to investigate the contribution of cysteine proteases to virulence mechanism of *E. histolytica *[19,20] and A2- protein in the survival of *L. (L.) donovani *amastigotes in macrophages [21]. Furthermore, episomal expression of sense and anti sense mRNA of *L. (L.) amazonensis *gp63using *Leishmania*- specific P6.5 vector provided evidence for a role both in binding of macrophages and the intracellular survival and replication [22]. Not surprisingly, *L. (L.) chagasi *amastigotes expressing *Ldccys2 *antisense mRNA has clearly down regulated the endogenous *Ldccys2 *mRNA levels (Figure 5). However, a small amount of *Ldccys2 *mRNA is still present in antisense transfectants, which may be due to the incomplete suppression, which is inherent to this approach. Infection of U937 macrophage cells with transfectants expressing *Ldccys2 *antisense mRNA showed a significant decrease in amastigote survival inside the macrophage cells, as compared to amastigotes expressing either sense *Ldccys2 *mRNA or the control plasmid (Figure 5). This is in agreement with the results obtained from *Ldccys2 *heterozygous knockout mutants, reaffirming the role of Ldccys2 in initiation of intracellular infection, and subsequent survival and multiplication of amastigotes. However, the actual effect of antisense mRNA expression may be greater, since reversal of antisense effects is expected from the release of the selective pressure necessary to avoid drug toxicity to macrophages [21]. This is especially true in the assay for intracellular survival and replication, since it requires prolonged period of incubation. The transient nature of the antisense suppression in the absence of selection prevented us from taking up animal studies that also require prolonged infection with these transfectants.

The out come of this study shows that *Ldccys2 *is expressed only in amastigote stage of the parasite. Both the heterozygous knockout mutants and antisense mRNA expression shows that Ldccys2 is important for infection of macrophages and survival of *L. (L.) chagasi *amastigotes inside the macrophage cells. Based on the data obtained in this study, there is a clear justification for targeting the *Ldccys2 *gene in *L (L.). chagasi *and carrying out further studies in animal models. Currently, we are working towards generating null mutants of *Ldccys2 *in *L. (L.) chagasi *and to further understand the role of this protein in the pathogenesis of *L. (L.) chagasi *parasites.

## Methods

### Parasite culture

*L. (L.) chagasi *strain MHOM/BR/74/PP75 promastigotes were grown in HOMEM, pH 7.4 (Gibco BRL) supplemented with 10% (v/v) heat inactivated calf serum, at 26°C. Promastigote cultures were seeded at 1 × 10^6^parasites/ml and harvested in logarithmic or stationary growth phase, as defined by cell concentration. *L. (L.) chagasi *promastigotes were transformed to axenic amastigotes using the standardized protocol as described earlier [29]. All the transfectants were periodically converted to amastigotes by infecting U937 macrophage cells in order to ensure the property of virulence.

### RNA isolation and northern hybridization

Total RNA was isolated using trizol (GibcoBRL) as recommended by the manufacturer. Promastigote RNA was obtained from logarithmic and stationary growth phase parasites of *L. (L.) chagasi*. To isolate amastigote RNA, human macrophage cell line (U937, ATCC) was infected with promastigote parasites, which were subsequently converted into intracellular amastigotes [29]. RNA (10 μg/lane) was separated on 1.2% (w/v) formaldehyde agarose gels and transferred onto Hybond N+ membrane (Amersham Pharmacia). Standard procedures were followed to perform Southern and Northern blot hybridizations. Using Quick Prime Labeling Kit (Amersham Pharmacia Biotech), probes representing specific region of *Ldccys2 *cDNA clone was labeled with [α^32^P] dCTP. The sense and antisense probes were generated by 5'end-labeling the specific sense and antisense oligonuecleotide primers with γ-^32^P ATP, using T4 polynucleotide kinase according to the standard protocol.

### Polyclonal antibody production against Ldccys2

Direct DNA immunization [30] was used to generate antibody against Ldccys2. Briefly, the pro-mature region of the gene was PCR amplified from the *Ldccys2 *cDNA clone using specific 5' (5'CAGACAGGATCCG CGGCCGCCATGGACGACTTCATTGCC 3') and 3' primers (5' GGCCGCGGATCCGCG GCCGCCTATGAGGTGTTGGAGTCGTC 3') and the PCR product was sub-cloned into *BamH*I site of pcDNA 3.1 (Invitrogen). After confirmation by sequencing the plasmid constructs were amplified in *E. coli *and DNA was isolated using endotoxin free maxi prep kit (Qiagen). 50 μg of plasmid DNA in 100 μl of saline was injected into the hind limb quadriceps muscles of BALB/c mice. All the mice were boosted after 14 days and tested for antibody response seven days after the boost. Mice with positive response were boosted one more time and after a week sera was collected and stored at -20°C for further use.

### Western blot analysis

Denaturing gel electrophoresis was carried out according to the standard protocols. Following SDS-PAGE, proteins were transferred onto to Hybond P membrane (Amersham Pharmacia Biotech) using semi-dry transblot apparatus (BioRad). Membranes were blocked for 2 hours with 5% (w/v) skim milk solution and probed overnight at 4°C with 1:200 dilution of anti-Ldccys2 antiserum. Membranes were washed three times with phosphate buffered saline containing 0.1% (v/v) Tween and incubated with 1:5000 dilution of anti-mouse horseradish peroxidase linked secondary antibody (Molecular Probes). Enhanced chemiluminescent kit (Amersham Pharmacia Biotech) was used for detection.

### Expression of cysteine protease genes

In order to express *Ldccys2 *cysteine protease gene, the coding region was amplified by PCR. The digested PCR product was cloned in-frame into unique NotI site of pIE1/153A.jhe (6hep) vector [31] and confirmed by sequencing. The fusion of the Cysteine proteases to the c-terminus of Juvenile hormone esterase (JHE) directs the secretion into the culture supernatant of transfected insect cells. Culture of insect cells and procurement of samples containing recombinant protein from transfected insect cells are described earlier [32]. Briefly, High Five insect cells (Invitrogen) were seeded into6-well culture plates (35 mm diameter) at a density of 5 × 10^5 ^cells/ml (2 ml/well) and transfected for 5 h with 0.5 ml of transfection solution containing 30 μg/ml lipofectin (Life Technologies) and 6 μg/ml total plasmid DNA in basal IPL-41 medium. Then the transfection solution was removed, the cells were rinsed with basal media and 2 ml IPL-41 + 10% FBS was added and incubated for sixty hours. The supernatant was then harvested and stored at -20°C for analysis. Enzyme activity was analyzed on gelatin SDS-PAGE containing 0.2% gelatin and 8% (w/v) acrylamide as described previously [17]. For inhibition studies, gels were pre-incubated with 20 μM E-64 (Sigma), a Cysteine protease specific inhibitor. Gels were stained with Coomassie Brilliant Blue for half hour and destained with 30% Methanol for 2 hours, to visualize the bands. Bands were photographed using Diamed apparatus (Bio-Rad).

### Ldccys2 gene disruption by homologous recombination

Gene replacement vector, *pXLdccys2-HygKO *was designed to replace 500 bp mature region of *Ldccys2 *coding region and constructed as outlined below (Figure 2b). The sense primer *Ldccys2 Hind*III (5' GCTCTCAAGCTTGCTCACGCATCCGCCGC-3') and the antisense primer *Ldccys2 *Xho I (5'GAAGGCCTCGAGCGAGCCGCACATTCCCTG-3') were used to amplify a 500 bp region from *Ldccys2 *cDNA and cloned between *Hind*III – *Xho*I sites in pX63-Hyg (kindly provided by Dr. S. M. Beverley) for the 5' end homologous recombination. A 450 bp 3' end homologous recombination fragment was amplified using the sense primer *Ldccys2 Sma*I (5'CCGGAGCCCGGGCCCACGGCGCTTGTGCAG-3') and antisense primer *Ldccys2 Bgl*II (5' ACTGTCAGATCTGCTGTGCGCCAGATCGCG-3') and cloned between *Sma*I and *Bgl*II sites of *pX63-Hyg*. The plasmid construct *pXLdccys2-HygKO *was confirmed by sequencing and digested with *Hind*III and *Bgl*II. The resulting 3.8 kb linear fragment containing hygromycin phospotransferase/dihydrofolate reductase-thymidylate synthase (*hyg/dhfr-ts*) gene sequence and the homologous recombination fragment was purified and used for transfection.

### Episomal expression of Ldccys2 specific sense and antisense mRNA

The *Ldccys2 *cDNA clone was isolated from an amastigote specific cDNA library of *L. (L.) chagasi*. *Ldccys2 *was earlier reported as promastigote specific cDNA clone [17]. However, it was further verified to be amastigote specific gene (18). The coding region (1 kb) of *Ldccys2 *was inserted at *BamH*I site of *Leishmania *specific vector P6.5 [22], a kind gift from Dr. K.P.Chang. Cloning was done in both sense and antisense orientations with reference to that of N-acetylglucosamine 1-phosphate transferase *(nagt)*, which was used as the selective marker for tunicamycin resistance. Plasmid constructs were confirmed by sequencing. P6.5 plasmid and the sense and antisense plasmid constructs were amplified in *E. coli *and isolated with a DNA maxi prep kit (Qiagen) and the purified DNA samples were used for transfection.

### DNA transfection

All the transfections were carried out using *L. (L.) chagasi *promastigotes. Briefly, 4 × 10^7 ^log phase promastigotes were pelleted, washed twice with phosphate buffered saline and resuspended in 0.4 ml of electroporation buffer in a 0.2 cm cuvette. Twenty-five microgram of P6.5 plasmid DNA constructs was used for episomal expression, whereas for homologous recombination 5 μg of linearized DNA fragment was transfected. DNA was electroporated using BioRad Gene Pulser Unit at 0.45 kV, capacitance of 25 μF and resistance of 250 ohms. Following electroporation, cells were resuspended in drug-free media for 24 hours. Parasites with P6.5 plasmid DNA constructs were selected for tunicamycin resistance at 5, 10 and 20 μg/ml tunicamycin (Sigma). Parasites transfected with DNA fragment for homologous recombination were selected for hygromycin B (GibcoBRL) resistance at 100 μg/ml.

### In vitro assay for intra macrophage survival

The U937 macrophage cells were infected with *L. (L.) chagasi *stationary promastigotes at a host to parasite ratio of 1:10 [33]. Briefly, U937 suspension cells (10 ^6 ^cells/ml) in 10 ml RPMI 1640 medium supplemented with 10% (v/v) heat inactivated fetal bovine serum, 2 mM L-glutamine and 50 μg gentamycin (Gibco BRL) were allowed to grow at 37°C for 3 days in 10% (v/v) CO_2_. The cells were then induced to adhere with 7.5 ng/ml of phorbol myristate acetate (PMA) and allowed to recover for 72 hours. The macrophage cells were then infected with stationary phase promastigotes and allowed to infect for 6 hours. The unbound parasites were washed away with RPMI medium and the infected macrophage cells were fed with fresh RPMI medium and left for 4 days. At every 12 hours interval up to 72 hours, cells were scraped and Diff-Quick stained slides were prepared for microscopic counting,

## Abbreviations

Hyg, hygromycin; JHE, juvenile hormone esterase; kb, kilobase; KO, knock-out; ORF, open reading frame; UTR, untranslated region

## Authors' contributions

VM carried out all the plasmid constructs for gene disruption and antisense mRNA inhibiton studies and performed the Southern and Northern blot analyses shown in Figures 1, 3, 4 & 6; wrote parts of the manuscript. ASK performed all the transfections, western blots and macrophage assays shown in Figures 1, 2, 4, 5, 6 and 7; wrote parts of the manuscript. LG supervised the experiments and gave laboratory support, and critically read the manuscript. All authors read and approved the final manuscript.
